# Causal relationship between inflammatory bowel disease and sex: a Mendelian randomization study

**DOI:** 10.3389/fendo.2025.1338701

**Published:** 2025-01-29

**Authors:** Kaiwen Wang, Yu Lou, Shunjie Tian, Zhihui Tao

**Affiliations:** ^1^ Department of Oncology, Seventh People’s Hospital of Shanghai University of Traditional Chinese Medicine, Shanghai, China; ^2^ Department of Renji Hospital, Shanghai Jiao Tong University School of Medicine, Shanghai, China; ^3^ Department of Shanghai University of Traditional Chinese Medicine, Shanghai, China

**Keywords:** sex hormone, IBD, Mendelian randomization, genome-wide association, biomarker

## Abstract

**Objective:**

The aim of this study was to investigate the bidirectional causal relationship between sex hormones and IBD through a two-sample bidirectional Mendelian randomization (MR) study.

**Methods:**

Based on Genome-Wide Association Study (GWAS) pooled data on SHBG, total testosterone, bioavailable testosterone, estradiol, and IBD in a European population, we performed two-sample bidirectional MR analyses using single nucleotide polymorphisms (SNPs) as instrumental variables. We used inverse variance weighting (IVW), weighted median, weighted mode, and MR-Egger to assess bidirectional causality between sex hormones and IBD.

**Results:**

There was no causal relationship between sex hormones and IBD in women (*P* > 0.05), and there was a causal and positive correlation between SHBG and testosterone and IBD in men.The OR for SHBG was 1.22 (95% CI: 1.09-1.37, *P* = 0.0004), and for testosterone was 1.20 (95% CI: 1.04-1.39, *P* = 0.0145).IBD did not significantly interact with female sex hormones but resulted in a decrease in SHBG (OR = 1.02, 95% CI: 1.00-1.04, *P* = 0.0195) and testosterone (OR = 1.01, 95% CI: 1.00 -1.02, *P* = 0.0200) in men.

**Conclusion:**

There is no causal relationship between female sex hormones and IBD, but male SHBG and testosterone are positively correlated with the risk of IBD and IBD promotes elevated levels of SHBG and testosterone in males, suggesting that sex hormones play different roles in IBD patients of different sexes.

## Introduction

Inflammatory bowel disease (IBD) encompasses Crohn’s disease (CD) and ulcerative colitis (UC), and its prevalence has been steadily increasing in recent years (1). The development of IBD is a complex process influenced by both environmental and genetic factors (2). Previous studies have reported a disparity in the prevalence of IBD between men and women, with a higher incidence observed in men (3). A meta-analysis further supports this finding, revealing a predominant male predominance among adult IBD patients (4). Recent studies have indicated that sex hormones possess the ability to modulate the progression of IBD and impact its development (5). Furthermore, individuals utilizing hormone replacement therapy, which includes sex hormones, have been observed to exhibit a reduced risk of developing IBD (6). IBD may also impact the production of sex hormones. A study revealed that adolescent boys with IBD had delayed puberty and growth, which showed improvement upon taking androgens (7, 8).

The progression of IBD has been shown to interfere with the regulation of hormone production in the body. For instance, a study demonstrated that mice with early-stage DSS-induced enterocolitis exhibited significantly smaller seminal vesicles and reduced levels of circulating androgens (9). Similarly, male patients with CD were found to have markedly lower serum levels of testosterone, estradiol, and sex hormone-binding globulin compared to healthy individuals (10). These findings highlight a potential link between sex hormones and IBD, suggesting reciprocal interactions. However, the precise causal relationship and underlying mechanisms remain poorly understood.

Mendelian randomization (MR) is an analytical method used to assess causality by utilizing genetic variants that are closely associated with exposure as instrumental variables (IVs) (11, 12). This method is advantageous in minimizing confounding factors, as the categorization of genetic variants occurs randomly at the time of conception and is independent of environmental influences. Now for sex hormones and IBD no one has studied them using Mendelian randomization. Therefore, in this study we aimed to investigate the potential bidirectional causal relationship between IBD and sex hormones by MR analysis of pooled data from two samples.

## Methods

### Data sources

All identified datasets involved in this study are publicly available from the ieu dataset (https://gwas.mrcieu.ac.uk/datasets) (13). Ethical approval was obtained for all original studies. The relevant ieu data used are shown in **Table 1**.

**Table 1:** The sources of GWAS data.

GWASID	Year	Trait	Sample size	Number of SNPs
ieu-b-4870	2020	Sex hormone binding globulin (SHBG)	214989	12321875
ieu-b-4871	2020	Sex hormone binding globulin (SHBG)	185221	12321875
ieu-b-4864	2020	Total Testosterone	199569	12321875
ieu-b-4865	2020	Total Testosterone	199569	12321875
ieu-b-4868	2020	Bioavailable Testosterone	184,205	12,321,875
ieu-b-4869	2020	Bioavailable Testosterone	180386	12,321,875
ieu-b-40	2018	Body mass index	681275	2336260
ieu-b-142	2019	Cigarettes smoked per day	249752	12003613
finn-b-K11_KELAIBD	2021	IBD patients in KELA-register		16380455

### Instrument selection

In order to effectively demonstrate causal effects, instrumental variables (IVs) used in MR analyses must meet three key conditions: (1) they must have a strong association with the exposure, (2) they must not be associated with any confounders, and (3) they must only influence the outcome through the exposure and not through other pathways (14). To select the instrumental variables (IVs), we followed the following criteria: (1) SNPs associated with exposure at the genome-wide motif significance threshold (*P* < 5 × 10^-8^) were considered as potential IVs; (2) We used the 1000 Genomes Project European samples data as a reference panel to identify SNPs with low linkage disequilibrium (LD) and R2 < 0.001 (aggregation window size = 10,000 kb), and only the SNPs with the lowest P-value were retained to ensure that each SNP is independent; (3) SNPs with a minor allele frequency (MAF) ≤ 0.01 were excluded to exclude rare variants; and (4) In cases where palindromic SNPs were present, we inferred the forward stranded alleles using allele frequency information; (5) SNPs associated with confounders (smoking, alcohol consumption, BMI, etc.) were removed using Phenoscanner. In addition, the strength of IVs was assessed using the F statistic (F = beta2/se2) for each SNP, and SNPs with F < 10 were excluded (15) to reduce bias from weak instrumental variables.

### Statistical analyses

In this study, we employed multiple methods, including Inverse Variance Weighting (IVW), Simple mode, MR Egger regression, weighted median, and weighted modeling, to investigate the potential causal relationship between sex hormones and IBD. Moreover, we utilized MR-PRESSO analysis to identify and mitigate horizontal pleiotropy by eliminating significant outliers. The heterogeneity of individual SNP effects was assessed using the Cochran Q test (16). Furthermore, to evaluate the causal association between IBD and sex hormones, we conducted a reverse MR analysis, employing the same methods and settings as the forward MR analysis. The estimates are expressed as odds ratios (ORs) with 95% confidence intervals (CIs), which indicate the average change in the outcome resulting from each exposure. These results only provide evidence of a causal effect between the exposure and the outcome, without any other interpretation.

The analyses were primarily conducted using the statistical software R (version 4.1.0). Various packages including TwoSampleMR (version 0.5.6), MendelianRandomization (version 0.6.0), MRPRESSO (version 1.0), and ggplot2 (version: 3.3.5) were utilized for data manipulation, graph creation, and reading.

## Results

### Causal relationship between female sex hormones and IBD

**Figure 1:**
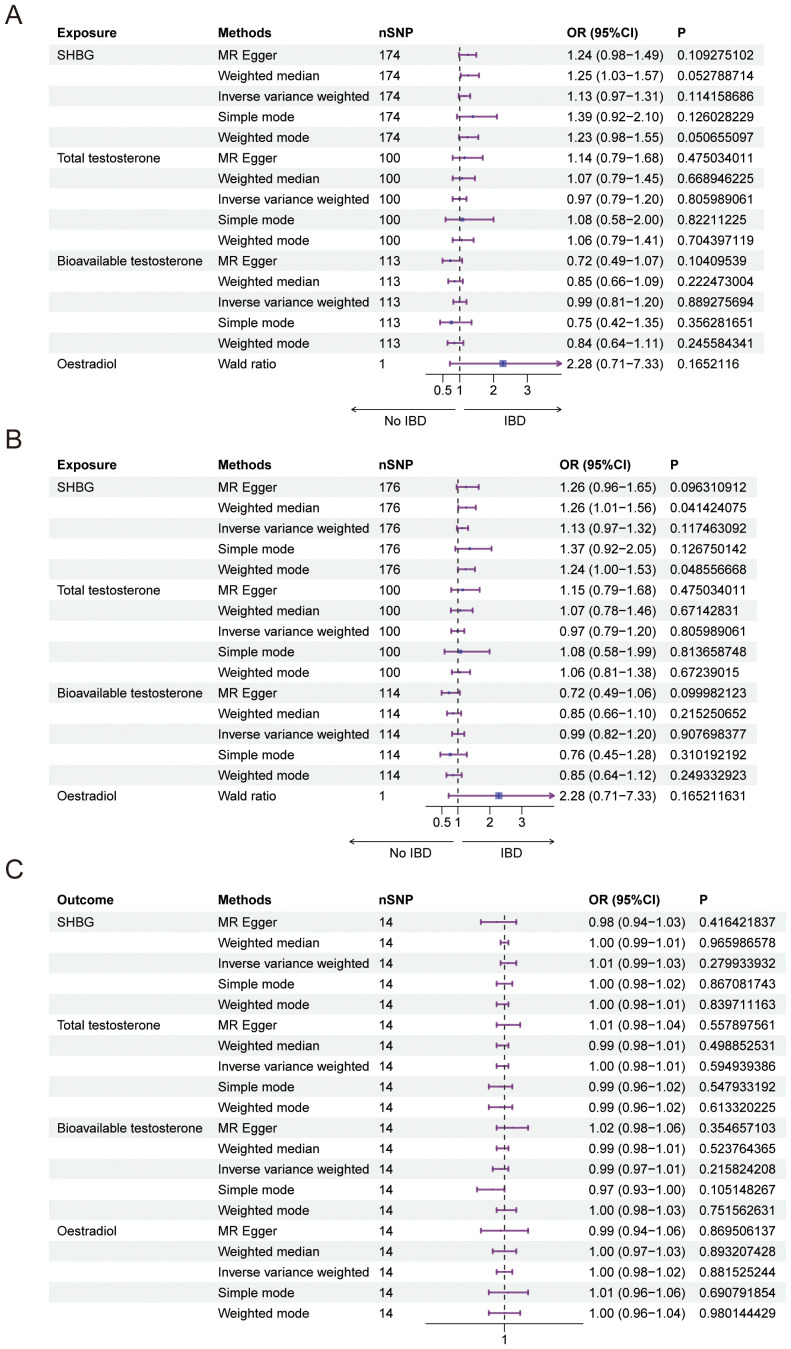
Causal relationship between female sex hormone and IBD. **(A)** Forest plot of the causal relationship between female sex hormones and IBD via univariate Mendelian randomization. **(B)** Forest plot of the causal relationship between female sex hormones and IBD by multivariate Mendelian randomization. **(C)** Forest plot of the causal relationship between IBD and female sex hormones. SHBG, Sex Hormone-Binding Globulin; nSNP, Number of Single Nucleotide Polymorphisms; OR (95% CI), Odds Ratio (95% Confidence Interval); MR Egger, Mendelian Randomization Egger Regression; IVW, Inverse Variance Weighted.

We conducted a Mendelian randomization (MR) analysis to investigate the relationship between female sex hormone levels (SHBG, total testosterone, bioavailable testosterone, and estradiol) and inflammatory bowel disease (IBD). After excluding factors such as horizontal pleiotropy and F>10, we identified a total of 174,100,113 and 1 SNPs for MR analysis, respectively. However, our findings did not reveal any statistically significant effect of SHBG, total testosterone, bioavailable testosterone, or estradiol on IBD in females (**Figure 1A**, **Table 2**). Furthermore, the funnel plot shows no bias (**Supplementary Figures 1A–C**) and removal-by-removal tests demonstrated that no individual SNP significantly influenced the robustness of our results, indicating the stability of our study (**Supplementary Figures 1D–F**).

**Table 2:** MR analysis of the causal relationship between female sex hormones and IBD.

Exposure	Methods	nSNP	OR (95%CI)	*P*
SHBG	MR Egger	174	1.24 (0.98-1.49)	0.1092751
	Weighted median	174	1.25 (1.03-1.57	0.05278871
	Inverse variance weighted	174	1.13 (0.97-1.31)	0.11415869
	Simple mode	174	1.39 (0.92-2.10)	0.12602823
	Weighted mode	174	1.23 (0.98-1.55)	0.0506551
Total testosterone	MR Egger	100	1.14 (0.79-1.68)	0.47503401
	Weighted median	100	1.07 (0.79-1.45)	0.66894623
	Inverse variance weighted	100	0.97 (0.79-1.20)	0.80598906
	Simple mode	100	1.08 (0.58-2.00)	0.82211225
	Weighted mode	100	1.06 (0.79-1.41)	0.70439712
Bioavailable testosterone	MR Egger	113	0.72 (0.49-1.07)	0.10409539
	Weighted median	113	0.85 (0.66-1.09)	0.222473
	Inverse variance weighted	113	0.99 (0.81-1.20)	0.88927569
	Simple mode	113	0.75 (0.42-1.35)	0.35628165
	Weighted mode	113	0.84 (0.64-1.11)	0.24558434
Oestradiol	Wald ratio	1	2.28 (0.71-7.33)	0.1652116

In our study, we investigated the causal relationship between sex hormones and IBD using multivariate Mendelian randomization. After accounting for confounding factors such as other sex hormones, BMI, and smoking, we did not find a statistically significant relationship between sex hormones and IBD in women (**Figure 1B**, **Table 3**). Furthermore, we conducted reverse Mendelian randomization to explore the causal association between IBD and female sex hormones, and our results also showed no statistically significant association between IBD and female sex hormones (**Figure 1C**, **Table 4**).

**Table 3:** Multivariate MR analysis of the causal relationship between female sex hormones and IBD.

Exposure	Methods	nSNP	OR (95%CI)	P
SHBG	MR Egger	176	1.26 (0.96-1.65)	0.09631091
	Weighted median	176	1.26 (1.01-1.56)	0.04142408
	Inverse variance weighted	176	1.13 (0.97-1.32)	0.11746309
	Simple mode	176	1.37 (0.92-2.05)	0.12675014
	Weighted mode	176	1.24 (1.00-1.53)	0.04855667
Total testosterone	MR Egger	100	1.15 (0.79-1.68)	0.47503401
	Weighted median	100	1.07 (0.78-1.46)	0.67142831
	Inverse variance weighted	100	0.97 (0.79-1.20)	0.80598906
	Simple mode	100	1.08 (0.58-1.99)	0.81365875
	Weighted mode	100	1.06 (0.81-1.38)	0.67239015
Bioavailable testosterone	MR Egger	114	0.72 (0.49-1.06)	0.09998212
	Weighted median	114	0.85 (0.66-1.10)	0.21525065
	Inverse variance weighted	114	0.99 (0.82-1.20)	0.90769838
	Simple mode	114	0.76 (0.45-1.28)	0.31019219
	Weighted mode	114	0.85 (0.64-1.12)	0.24933292
Oestradiol	Wald ratio	1	2.28 (0.71-7.33)	0.16521163

**Table 4:** MR analysis of the causal relationship between IBD and female sex hormones.

Outcome	Methods	nSNP	OR (95%CI)	P
SHBG	MR Egger	14	0.98 (0.94-1.03)	0.41642184
	Weighted median	14	1.00 (0.99-1.01)	0.96598658
	Inverse variance weighted	14	1.01 (0.99-1.03)	0.27993393
	Simple mode	14	1.00 (0.98-1.02)	0.86708174
	Weighted mode	14	1.00 (0.98-1.01)	0.83971116
Total testosterone	MR Egger	14	1.01 (0.98-1.04)	0.55789756
	Weighted median	14	0.99 (0.98-1.01)	0.49885253
	Inverse variance weighted	14	1.00 (0.98-1.01)	0.59493939
	Simple mode	14	0.99 (0.96-1.02)	0.54793319
	Weighted mode	14	0.99 (0.96-1.02)	0.61332023
Bioavailable testosterone	MR Egger	14	1.02 (0.98-1.06)	0.3546571
	Weighted median	14	0.99 (0.98-1.01)	0.52376436
	Inverse variance weighted	14	0.99 (0.97-1.01)	0.21582421
	Simple mode	14	0.97 (0.93-1.00)	0.10514827
	Weighted mode	14	1.00 (0.98-1.03)	0.75156263
Oestradiol	MR Egger	14	0.99 (0.94-1.06)	0.86950614
	Weighted median	14	1.00 (0.97-1.03)	0.89320743
	Inverse variance weighted	14	1.00 (0.98-1.02)	0.88152524
	Simple mode	14	1.01 (0.96-1.06)	0.69079185
	Weighted mode	14	1.00 (0.96-1.04)	0.98014443

### Causal relationship between male sex hormones and IBD

**Figure 2:**
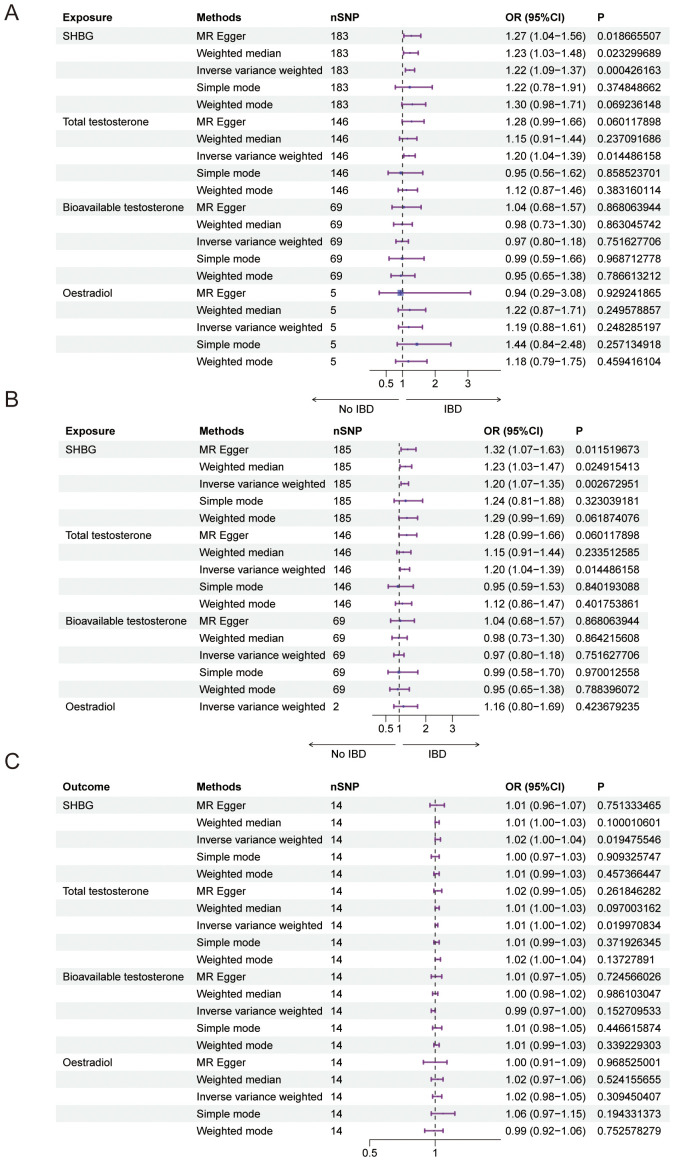
Causal relationship between male sex hormone and IBD. **(A)** Forest plot of the causal relationship between male sex hormones and IBD via univariate Mendelian randomization. **(B)** Forest plot of the causal relationship between male sex hormones and IBD by multivariate Mendelian randomization. **(C)** Forest plot of the causal relationship between IBD and male sex hormones. SHBG, Sex Hormone-Binding Globulin; nSNP, Number of Single Nucleotide Polymorphisms; OR (95% CI), Odds Ratio (95% Confidence Interval); MR Egger, Mendelian Randomization Egger Regression; IVW, Inverse Variance Weighted.

We conducted a Mendelian randomization (MR) analysis to examine the relationship between male sex hormone levels (SHBG, total testosterone, bioavailable testosterone, and estradiol) and inflammatory bowel disease (IBD). We excluded factors such as horizontal pleiotropy and F>10, resulting in a total of 183,146,69 SNPs and 5 SNPs for MR analysis, respectively. Our findings revealed a positive and causal correlation between SHBG and total testosterone with IBD. The odds ratio (OR) for the IVW analysis method was 1.22 (95% CI: 1.09-1.37, P = 0.0004) for SHBG and 1.20 (95% CI: 1.04-1.39, P = 0.0145) for SHBG. However, bioavailable testosterone and estradiol did not show statistical significance (**Figure 2A**, **Table 5**). Additionally, the funnel plot shows no bias (**Supplementary Figures 2A–C**) and the one-by-one method did not identify any single SNP that significantly influenced the results, indicating the stability of our findings (**Supplementary Figures 1D–F**).

**Table 5:** MR analysis of the causal relationship between male sex hormones and IBD.

Exposure	Methods	nSNP	OR (95%CI)	*P*
SHBG	MR Egger	183	1.27 (1.04-1.56)	0.01866551
	Weighted median	183	1.23 (1.03-1.48)	0.02329969
	Inverse variance weighted	183	1.22 (1.09-1.37)	0.00042616
	Simple mode	183	1.22 (0.78-1.91)	0.37484866
	Weighted mode	183	1.30 (0.98-1.71)	0.06923615
Total testosterone	MR Egger	146	1.28 (0.99-1.66)	0.0601179
	Weighted median	146	1.15 (0.91-1.44)	0.23709169
	Inverse variance weighted	146	1.20 (1.04-1.39)	0.01448616
	Simple mode	146	0.95 (0.56-1.62)	0.8585237
	Weighted mode	146	1.12 (0.87-1.46)	0.38316011
Bioavailable testosterone	MR Egger	69	1.04 (0.68-1.57)	0.86806394
	Weighted median	69	0.98 (0.73-1.30)	0.86304574
	Inverse variance weighted	69	0.97 (0.80-1.18)	0.75162771
	Simple mode	69	0.99 (0.59-1.66)	0.96871278
	Weighted mode	69	0.95 (0.65-1.38)	0.78661321
Oestradiol	MR Egger	5	0.94 (0.29-3.08)	0.92924187
	Weighted median	5	1.22 (0.87-1.71)	0.24957886
	Inverse variance weighted	5	1.19 (0.88-1.61)	0.2482852
	Simple mode	5	1.44 (0.84-2.48)	0.25713492
	Weighted mode	5	1.18 (0.79-1.75)	0.4594161

In addition, we also used multivariate Mendelian randomization to explore the causal relationship between sex hormones and IBD. After excluding the effects of other sex hormones, BMI and smoking as confounders, we found that, consistent with the results of the univariate Mendelian randomization, the OR for SHBG in men was 1.20 (95%CI: 1.07-1.35, *P* = 0.0027), and that the OR for total testosterone in men was 1.20 (95%CI: 1.04-1.39, *P* = 0.0144), and bioavailable testosterone and estradiol were not statistically significant (**Figure 2B**, **Table 6**). We also performed reverse Mendelian randomization to investigate the causal association between IBD and sex hormones and demonstrated a significant association between IBD and SHBG and total testosterone in men, with an OR of 1.02 (95% CI: 1.00-1.04, P=0.0195) for IBD on SHBG in men and an OR of 1.01 (95% CI: 1.00-1.02, P=0.0200), and was not statistically significant for bioavailable testosterone versus estradiol in men (**Figure 2C**, **Table 7**).

**Table 6:** Multivariate MR analysis of the causal relationship between male sex hormones and IBD.

Exposure	Methods	nSNP	OR (95%CI)	*P*
SHBG	MR Egger	185	1.32 (1.07-1.63)	0.01151967
	Weighted median	185	1.23 (1.03-1.47)	0.02491541
	Inverse variance weighted	185	1.20 (1.07-1.35)	0.00267295
	Simple mode	185	1.24 (0.81-1.88)	0.32303918
	Weighted mode	185	1.29 (0.99-1.69)	0.06187408
Total testosterone	MR Egger	146	1.28 (0.99-1.66)	0.0601179
	Weighted median	146	1.15 (0.91-1.44)	0.23351259
	Inverse variance weighted	146	1.20 (1.04-1.39)	0.01448616
	Simple mode	146	0.95 (0.59-1.53)	0.84019309
	Weighted mode	146	1.12 (0.86-1.47)	0.40175386
Bioavailable testosterone	MR Egger	69	1.04 (0.68-1.57)	0.86806394
	Weighted median	69	0.98 (0.73-1.30)	0.86421561
	Inverse variance weighted	69	0.97 (0.80-1.18)	0.75162771
	Simple mode	69	0.99 (0.58-1.70)	0.97001256
	Weighted mode	69	0.95 (0.65-1.38)	0.78839607
Oestradiol	Inverse variance weighted	2	1.16 (0.80-1.69)	0.42367924

**Table 7:** MR analysis of the causal relationship between IBD and male sex hormones.

Outcome	Methods	nSNP	OR (95%CI)	*P*
SHBG	MR Egger	14	1.01 (0.96-1.07)	0.75133347
	Weighted median	14	1.01 (1.00-1.03)	0.1000106
	Inverse variance weighted	14	1.02 (1.00-1.04)	0.01947555
	Simple mode	14	1.00 (0.97-1.03)	0.90932575
	Weighted mode	14	1.01 (0.99-1.03)	0.45736645
Total testosterone	MR Egger	14	1.02 (0.99-1.05)	0.26184628
	Weighted median	14	1.01 (1.00-1.03)	0.09700316
	Inverse variance weighted	14	1.01 (1.00-1.02)	0.01997083
	Simple mode	14	1.01 (0.99-1.03)	0.37192634
	Weighted mode	14	1.02 (1.00-1.04)	0.13727891
Bioavailable testosterone	MR Egger	14	1.01 (0.97-1.05)	0.72456603
	Weighted median	14	1.00 (0.98-1.02)	0.98610305
	Inverse variance weighted	14	0.99 (0.97-1.00)	0.15270953
	Simple mode	14	1.01 (0.98-1.05)	0.44661587
	Weighted mode	14	1.01 (0.99-1.03)	0.3392293
Oestradiol	MR Egger	14	1.00 (0.91-1.09)	0.968525
	Weighted median	14	1.02 (0.97-1.06)	0.52415566
	Inverse variance weighted	14	1.02 (0.98-1.05)	0.30945041
	Simple mode	14	1.06 (0.97-1.15)	0.19433137
	Weighted mode	14	0.99 (0.92-1.06)	0.75257828

## Discussion

In this study, we investigated the bidirectional causal relationship between sex hormones and IBD by Mendelian randomization. We found that there was no causal relationship between sex hormones and IBD in women, whereas SHBG and total testosterone were positively correlated with each other and IBD in men, and the reliability of our conclusions was further demonstrated by sensitivity analysis.

IBD is a chronic disease (17) and it is worth noting that there are sex differences in the risk of developing IBD (18, 19) A previous study found that younger women had a lower incidence of IBD than men, but women over 35 had higher IBD compared to men, so we hypothesized that estrogen may be playing a role (20) There have been studies demonstrating an association between estrogen and IBD, and one mouse experiment demonstrated that estradiol may be involved in the progression of IBD, with her mediating a less severe degree of IBD in female mice and a better ability to recover (21). In addition, other studies have shown that estrogen receptors play an important role in the progression of IBD (22).

Total testosterone refers to the combined concentrations of protein-bound and unbound testosterone in the bloodstream. Available testosterone, on the other hand, represents the portion of circulating testosterone that is not bound to sex hormone-binding globulin (SHBG). SHBG is produced by the liver and has a strong attraction to testosterone, with both substances playing a crucial role in the functioning of living organisms (23). Some studies have demonstrated that SHBG-bound testosterone is distributed differently in men and women (23, 24) A significant association between SHBG and testosterone in gastrointestinal disorders has been observed in various studies. A meta-analysis conducted on the UK Biobank dataset revealed that SHBG and testosterone were found to be linked with an increased risk of colorectal cancer specifically in men, but no statistically significant association was observed in women (25, 26). This is consistent with our finding that SHBG and testosterone are associated with IBD risk only in men. Mechanistically, SHBG can mitigate oxidative stress by modulating the expression of endogenous antioxidant enzymes, including SOD1, CAT, and GPx. This regulation reduces the intracellular accumulation of ROS, ultimately alleviating inflammation (27). Furthermore, evidence suggests that SHBG also influences adipose tissue metabolism, contributing to the suppression of inflammatory responses (28). In addition, testosterone treatment induces an increase in pro-inflammatory cytokines which in turn affects the progression of inflammation (29). These findings highlight the significant roles of SHBG and testosterone in the pathogenesis of IBD, particularly in men.

It has been shown that estrogens play a protective role in the gastrointestinal tract (30), with estradiol (E2) being the most biologically active, and that the high binding affinity of SHBG for both estrogens and E2 leads to a decrease in the biological activity of E2 (31). There are several factors that affect SHBG: estrogen can increase SHBG levels; testosterone can decrease SHBG levels. Therefore, we performed multivariate Mendelian randomization to exclude the effects of other estrogens and found that SHBG is elevated for the risk of IBD in men. In addition to this, we performed reverse Mendelian randomization and found that IBD also leads to altered SHBG versus testosterone levels in men. A study showing that IBD affects sex hormone production in boys is consistent with our study (7, 8). Additionally, a cross-sectional study demonstrated that IBD severity significantly impacts sex hormone levels in an East Asian male cohort (32). Correspondingly, data from a Chinese population reveal that testosterone and androstenedione levels are inversely related to inflammatory markers (33). These findings suggest a linkage between sex hormones and IBD that extends beyond European demographics to include various ethnic groups.

The present study used bidirectional two-sample Mendelian analysis to explore the bidirectional causal relationship between sex hormones and IBD has the following advantages: first, it can exclude the bias caused by confounding factors. Second, the huge sample size of GWAS can ensure the correctness of the results.

The study has a few limitations that should be considered. Firstly, the population in this study was limited to individuals of European ancestry, and it is important to acknowledge that there may be genetic differences among different ethnic groups. Secondly, while this study establishes a causal relationship between sex hormones and IBD, it does not provide a detailed explanation of the specific molecular mechanisms involved. Further verification through functional experiments is necessary to gain a deeper understanding of these mechanisms. Although adjustments were made for certain confounders, other unknown factors may still influence IBD. Further research will be required to address these potential limitations in future studies.

In conclusion, we found that SHBG and total testosterone were positively correlated with the risk of IBD in men. In addition, IBD was also positively correlated with SHBG versus total testosterone in men and they may have a mutually reinforcing relationship. In addition, further studies on different populations are needed due to ethnospecificity. Our study indicates that SHBG and total testosterone could serve as potential biomarkers for guiding the management of IBD. These findings propose a basis for their application in tailoring treatment strategies and optimizing patient care in IBD management.

## Data Availability

The datasets presented in this study can be found in online repositories. The names of the repository/repositories and accession number(s) can be found in the article/**Supplementary Material**.
