# Supplementary material for: Causal relationship between inflammatory bowel disease and sex: a Mendelian randomization study
Source: Front Endocrinol (Lausanne). 2025 Jan 29;16:1338701. doi: 10.3389/fendo.2025.1338701 (PMC11813775; doi:10.3389/fendo.2025.1338701)
Supplement: Supplementary file 1 [file DataSheet1.pdf]

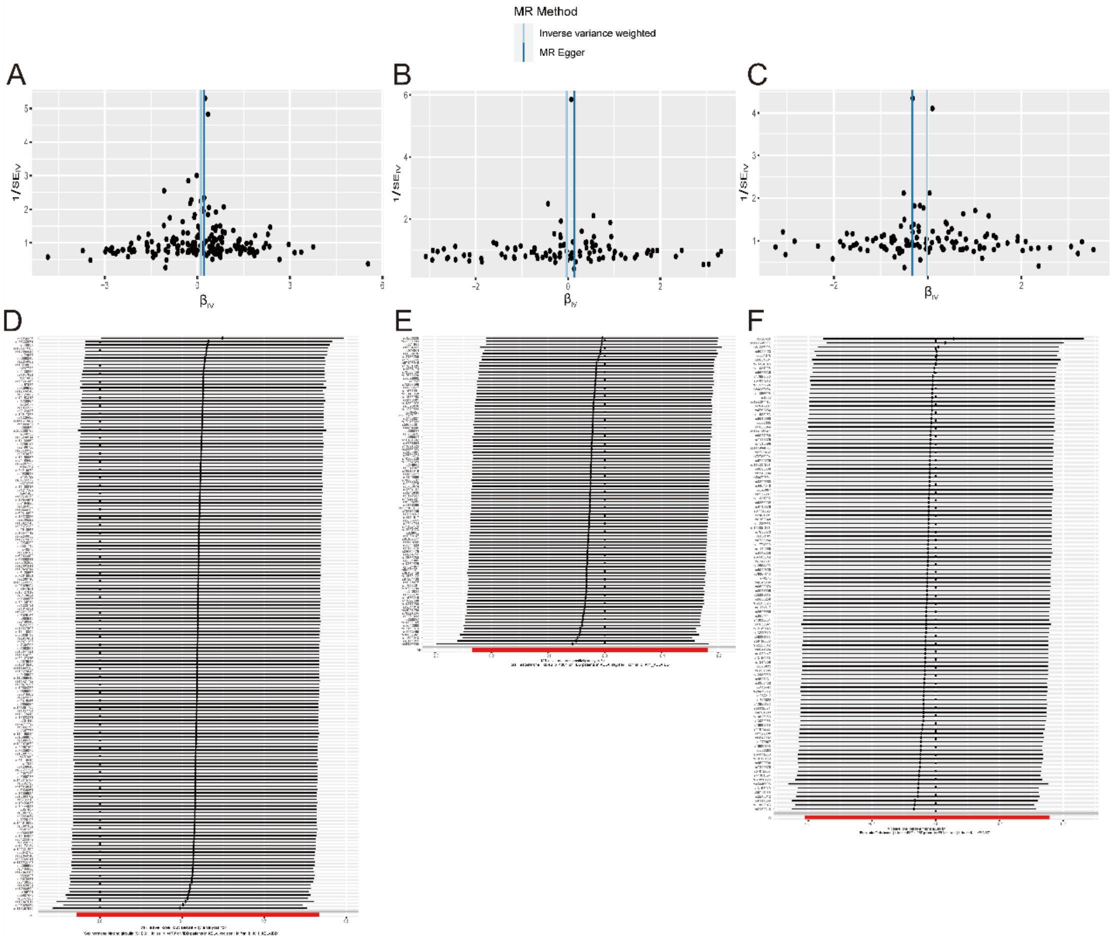

Supplementary Figure 1. Causal relationship between female sex hormone and IBD. (A-C) Funnel plot of the causal relationship between female sex hormone and IBD. (D-F) Leave-one-out plot of the causal relationship between female sex hormone and IBD.

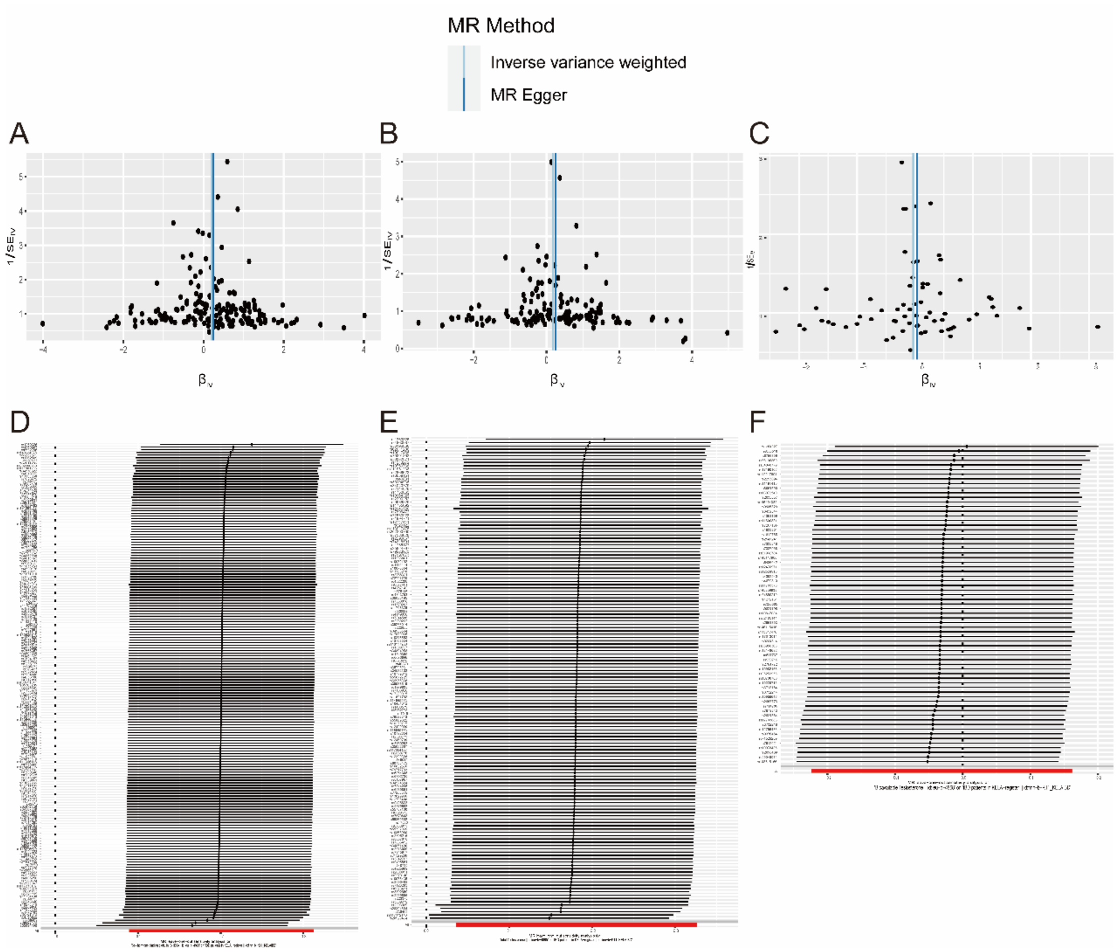

Supplementary Figure 2. Causal relationship between male sex hormone and IBD. (A-C) Funnel plot of the causal relationship between male sex hormone and IBD. (D-F) Leave-one-out plot of the causal relationship between male sex hormone and IBD.
